# Running a journal club in 2020: reflections and challenges

**DOI:** 10.1192/bjb.2020.121

**Published:** 2020-11-13

**Authors:** Isabel Mark, Motaz Sonbol, Cyrus Abbasian

**Affiliations:** 1 Springfield Hospital, South West London and St George’s Mental Health NHS Trust, London, UK; 2 St George's University of London, UK

**Keywords:** Journal club, online, engagement, post-graduate education, COVID-19

## Abstract

The online environment brings both challenges and opportunities. The skills learned in journal clubs remain highly relevant where the ability to critique rapidly generated information and apply evidence to patient care is vital. Creativity and flexibility are needed to ensure that learners’ needs are met and efforts are made to involve those who may not be naturally drawn to online environments. This article explores how journal clubs have been approached in the past, both in person and more recently online, considers techniques for maintaining engagement in online teaching and proposes new approaches for future journal clubs.

The Royal College of Psychiatrist’s curriculum for core trainees requires the development of critical appraisal knowledge, skills and habits for practising evidence-based medicine (EBM).^1^ Since 1999, critical appraisal skills have been assessed in MRCPsych written examinations.^2^ It is accepted that attending a journal club offers trainees a key opportunity to learn and practise critical appraisal skills. Attending and presenting at a journal club are recommended for all trainees, being a workplace-based assessment (WPBA) for core trainees within their curriculum and required for annual review of competency progression (ARCP).^1^ Monitoring of attendance and reinforcement of its importance are likely to vary within UK deaneries and internationally.

## Journal clubs of the past

Doctors assembling to review current literature has been recorded as far back as 1875.^3^ However, recent difficulties in engaging doctors in journal clubs have been discussed.^2^ In 2009, Agell wrote of a ‘journal club syndrome,’ in which he reported an observed correlation between reported ‘mishaps’ preventing trainees from engaging in the learning process and the timing of their scheduled presentations.^4^ This somewhat cynical view highlights the long-standing anxieties and struggles with engagement that trainees might face.

In 2008, Deenadayalan et al recommended establishing an overarching goal for a journal club which is reviewed and agreed by participants, recording and expecting attendance, establishing sustainable leadership with access to a statistical expert as needed and providing incentives such as food.^5^ Other aspects, such as article choice, are also specified. In psychiatry, Swift built on these strategies, highlighting the importance of structure, clear aims, a social aspect for the group and discussing selection of the facilitator (with critical appraisal and teaching skills but also able to provide enthusiasm and approachability for more junior colleagues).^2,6^ Moving away from the traditional format by introducing ‘evidence-based journal clubs’ has been suggested.^2,7^ Such sessions would address a specific clinical problem by referring to available literature.

## Journal clubs online: what we know so far

Although the COVID-19 pandemic resulted in a rapid move to online learning, journal clubs have existed in the virtual world for some time. These can take the form either of synchronous learning, when a teacher is present at the same time as students, or of asynchronous learning, when a teacher directs study but is not present when the learning takes place.

Many asynchronous options for journal review have been developed, supporting the face-to-face meetings. MacLaren et al have discussed the internet possibilities, using social media platforms such as Facebook, Twitter handles and Mentimeter as a means to widen potential journal club participation and support learning.^8^ Other e-sources are available for accessing mental health information, an example being the Mental Elf, which has used blogs, social media and gamification to incentivise users.^9^

There has been a recent surge of asynchronous Twitter journal clubs,^10–13^ in which participants contribute via tweets over a specified time period (typically between 1 h and 1 week), providing free participation, open journal access, time efficiency and a diverse international forum for discussion. Instructions for those wishing to set up an asynchronous journal club can involve a number of steps, including incorporating other social media platforms and considering the most convenient time for its target audience, to make their establishment accessible to all.^14^ Plante et al note that content may be unregulated and that some might struggle to learn from reviewing past discussions, and suggest that Wiki Journal Club (a website that allows collaboration in writing summaries, critical analysis and reviews of chosen papers, similar to Wikipedia) overcomes these barriers by providing editorial and professional moderation, easily referenced and referred to.^15^ MacRae et al found a significant improvement in critical appraisal skills among participants of their surgical journal club, compared with a control group, but acknowledged that those involved in a Twitter journal club are likely to be more motivated than the general surgeon population.^10^

Synchronous online journal clubs have had less exploration. Among Lin & Sherbino's suggested steps for setting up an online journal club is the added possibility of ‘live’ video panel discussions using technology such as Google ‘Hangouts on Air’ and YouTube.^16^ More recently, the *BJPsych* has collaborated with university psychiatric societies to help successfully launch live virtual journal clubs, with student presentations followed by expert-panel discussion.^17^ A similar approach has been followed with dental trainees, highlighting the benefits of accessibility, ease of interaction and effective learning.^18^

Although additional online options may increase the potential for journal club participation, they are unlikely to suit all trainees. Twitter is the most popular form of social media communication among healthcare professionals,^19^ but the exact prevalence of use among doctors, or more specifically trainees, is to our knowledge not reported. If medical education becomes dependent on social media platforms, the risk is that some will be alienated or simply not reached.

## Techniques for maintaining engagement with online learning

Difficulties in engaging learners online are a recognised challenge for educators; traditional face-to-face teaching methods cannot simply be adopted in the online environment.^20^ Additional considerations are needed (Box 1).

Box 1 Instructor considerations for maximising engagement in online learning

- Consider three domains: cognitive, affective and managerial^21^
- Existing face-to-face options cannot simply be replicated online; a new approach is needed^20^
- Initial resistance is to be expected and learners may need additional support at the outset^24^
- Clinical scenarios improve perceived relevance/authenticity^24^
- Consider the diversity of the group and individual learner needs and preferences^25,26^

Coppola et al proposed that the online instructor's role has three domains: cognitive, affective and managerial, achieving a balance between imparting information effectively, communicating approachability, enthusiasm and intimacy, and being an effective organiser and administrator.^21^ A recent Best Evidence in Medical Education (BEME) review describes techniques used by educators to maximise student engagement online, necessitated by COVID-19.^22^ These include the need for effective organisation and structure, and varied options for student interaction, such as using online chat features, polling, hand-raising and breakout rooms.^22^ Warren et al argue the need for flexibility in this process, incorporating different learning modalities, considering technical resources and support.^23^ Engagement can still be delayed unless a learner is willing to change habits and thus suspend their disbelief in a new approach, with additional support and time needed for them to do so.^24^

Online learning is often more effective when activities are ‘authentic’, using complex clinical scenarios and tasks to increase the perceived level of relevance for the learner.^24^ In the current climate, using cases or studies involving COVID-19 specifically might assist with this.

One must consider all students rather than just the most vocal or communicative.^25^ Haggis proposes that teachers address student diversity by shifting their approach away from the struggling learners towards a more dynamic stance, considering the overall student–teacher interactive process.^25^ Problematic areas need to be addressed, including acceptance of the wide range of students’ experience, motives, interaction and communication preferences. The instructor could consider using diverse examples when setting problems/tasks and fostering social relationships within the group, while still respecting the student's own responsibility for driving their learning and seeking help.^26^ Forcing some students to contribute, when not part of a shared collaborative process, could endanger their autonomy and motivation.^27^

## Recent learning following COVID-19 restrictions

In 2020, since the COVID-19 pandemic, psychiatry training has depended on online technology to replace all face-to-face journal club meetings. Within months, our use of platforms such as Zoom and Microsoft Teams became the ‘new normal’ and the only option for teaching and networking. Although some participants were already familiar with the array of online options, many may not have been and could still be struggling to keep up.

Within South West London and St George's Mental Health NHS Trust, we have continued to run a weekly journal club using the virtual platform of Microsoft Teams, supported by a WhatsApp group to offer further discussion as needed. Efforts were made to boost the effectiveness of the journal club by recruiting additional higher trainees to support the sessions, increasing the level of support offered to the lead presenter and making particular efforts during journal club meetings to encourage all to contribute. Although the numbers in the virtual room have been noticeably higher than those in face-to-face meetings before March 2020 – up to two or three times as many – engagement levels have been difficult to measure, with many of those present in the virtual room not taking an active part. In the absence of visual cues, with most turning off their screen camera and not using the ‘chat’ function, we struggle to assess who is actively engaging in the group and who finds it useful. Several trainees have commented on missing the social aspect of the group.

As it has become unclear how long COVID-19 restrictions will continue, morale has declined. The initial high level of discussion has not been maintained; not all trainees have appeared confident to engage, either by speaking openly, using the written chat/Q&A function or through the WhatsApp group. This experience is, of course, likely to vary between different hospitals, trusts and deaneries, depending on the confidence of trainees, as well as the ethos and culture of the organisation.

Reflections and feedback from trainees are continuously sought and contribute to future planning (Box 2), but we are aware that those most disengaged from the process are less likely to communicate their needs.

Box 2 Lessons learned from recent experience

- Assessing and maintaining engagement in online forums is a challenge
- Uncertainty about the future can be unsettling and disempowering for some trainees
- Trainees feel a loss of the social aspect of journal clubs
- Learning and training opportunities have been threatened by clinical workload, technological limitations and noisy surroundings
- Facilitator encouragement, support and continuity are critical
- Trainees appear to appreciate a flexible approach, such as presenting articles in pairs, structuring their session in varied ways and focusing on different aspects of the paper. Some are interested in statistical methods and details, whereas others prefer to focus on how the research relates to previous and future research or how they can make use of the data in their practice

## Journal clubs of the future

Journal clubs continue to be a valued part of psychiatry training, offering a key opportunity to learn and practise critical appraisal skills, presenting skills, critical thinking and structured discussion. There is no current alternative within the RCPsych curriculum that offers these opportunities, and journal club remains a WPBA for core trainees.^1^ The current pandemic reminds us how critical evidence-based medicine is to our practice, with a flurry of new research on COVID-19, and the need to recognise how new research should be integrated into practice. The transition to online journal clubs now requires a new set of organiser and administrator skills, which is a challenge in some departments. Even before 2020, journal clubs were struggling with participant engagement.^2,6^ Online journal clubs might be embraced by those with research or education focus; but there is a high risk that others could feel excluded. Others might be starting to get online fatigue.

Considering techniques for effective engagement in online teaching can enhance our ability to facilitate journal clubs.^20–23^ The use of clinical problems and specific tasks, similar to the previously introduced evidence-based journal clubs, may provide additional relevance and authenticity for learners.^6,7,24^ Considering the diverse range of learner needs and preferences is advised.^25^ The online platform potentially supports educators in managing diversity by improving accessibility, in particular for those shielding or working on a different clinical site. Publicising and encouraging the use of additional asynchronous learning resources^8,9^ such as Twitter journal clubs to support sessions^14^ is worthwhile.

When running future synchronous online journal clubs, instructors need to transform their approach, not only in offering appropriate guidance and knowledge, but also in helping to establish an approachable atmosphere, optimising the potential for discussion and collaboration.^22^ Trainee uncertainty about the future and feelings of social loss need to be acknowledged, as does the consideration of variability in resources and skills, with limited availability of functioning computers for some. Trainees have fed back that they appreciate additional guidance on presentation style, paper choice and additional resources. They have expressed a preference for flexibility, not defining the style of presentation and allowing the option of multiple communication forms (speaking in person, typing in the written ‘chat’, as well as using WhatsApp and other social media platforms). A challenge of measuring active participation remains, as the administrative resources required to record contributions might not be feasible within all training programmes. Forcing contribution could endanger trainee autonomy,^26^ although it should be recognised that it is an expected and important part of the trainee curriculum.^1^

This article can only be the start of a more complex discussion and re-evaluation of the function, purpose and delivery of online journal clubs and the part they play in the psychiatry curriculum, as well as considering the level of support provided to trainees and by whom. Collaboration and discussion between professional educators in neighbouring training programmes will help explore and expand the wider networking potential of journal clubs.

## About the authors

**Isabel Mark** is a Specialty Trainee Year 5 (ST5) general adult registrar at South West London and St George's Mental Health NHS Trust and a clinical teaching fellow and honorary clinical lecturer at St George's University of London, UK. **Motaz Sonbol** is an ST5 general adult registrar at South West London and St George's Mental Health NHS Trust. **Cyrus Abbasian** is a consultant psychiatrist at South West London and St George's Mental Health NHS Trust and an honorary senior lecturer at St George's University of London.

## Supplementary material

For supplementary material accompanying this paper visit https://doi.org/10.1192/bjb.2020.121. click here to view supplementary material
